# Fellow-Workers and Their Doings

**Published:** 1914-06-27

**Authors:** 


### ROUND THE WORLD.

### Fellow-Workers and their Doings.

[The Editor Invites Early Information and Contributions Marked "Fellow-Workers."]

Dr. J. G. Porter Phillips, M.D., M.R.C.P., who is to be congratulated on having been elected resident physician and medical superintendent at Bethlem Royal Hospital, was formerly a student at University College, Gower Street, and at Guy's Hospital. Qualifying in 1907 and taking the M.D. Lond. three years later, Dr. Phillips thus obtains an important lunacy post very early in his career. He has, of course, been serving at "Bethlem" as senior assistant physician, previous to which he was house physician there. Dr. Phillips was awarded the Gaskell Gold Medal in psychological medicine three years ago, and has carried out interesting observations on the treatment of mental disease.

Dr. Ralph Brown, who succeeds Dr. Phillips as senior resident assistant physician at "Bethlem," has been serving that institution as third in command. Qualifying from Westminster Hospital some eight years ago, Dr. Brown held resident appointments there, and then proceeded to the well-known "Moorcroft" private asylum at Hillingdon. He also is a member of the London Royal College of Physicians.

Dr. Maurice A. Cassidy, who has just been selected for the post of physician to the Metropolitan Police Force, is a well-known West End consultant attached to St. Thomas's Hospital. Dr. Cassidy, who is an M.D. Cantab., received the distinction of being made an F.R.C.P. Lond. comparatively early in his career, and has done much excellent work in his appointments of physician to out-patients, Salter's Research Fellow, and director of the electro-cardiographic department at St. Thomas's.

Dr. Howard Humphris, who presided recently at the Brussels Medical Graduates' Association's dinner held in the White City, is as well-known in America as in this country as a scientific exponent of electro-therapy, and was formerly a leading medical man in the republic of Hawaii. Dr. Humphris took the M.D. Brux., with honours, in 1895, the same year as he qualified M.R.C.S., L.R.C.P., and subsequently became elected a Fellow of the Royal College of Physicians of Edinburgh. He has done much to develop the use of static electricity and the use of the Bergonié apparatus in London, and is at the present time President of the American Electro-therapeutic Association.

Some important researches on the question as to whether infection by malaria will occasion a positive Wassermann reaction have been carried out by Dr. William Fletcher in the Malay States. Communicating his recent observations to the *Lancet* (June 13, 1914), this investigator reports that if the method of carrying out the Wassermann reaction associated with the names of Browning, Cruickshank, and McKenzie are carried out, the presence of malarial parasites in the blood does not give rise to a positive result. Dr. Fletcher, who is an M.D. Cantab., studied medicine at St. Mary's, and now holds the appointment of pathologist to the Medical Research Institute at Kuala Lumpur in the Federated Malay States.

The new bacteriologist at the Manchester Royal Eye Hospital, Mr. Arnold Renshaw, has qualified both in medicine and dentistry. As a dental graduate of the Manchester University as well as an M.B., B.S. Lond., Dr. Renshaw has been occupied with a good deal of scientific work at various Manchester institutions.

An interesting report on the clinical features of an epidemic of cerebro-spinal meningitis in the Bristol district has just been drawn up by Dr. J. Mitchell Clarke and Dr. J. Odery Symes, both of whom are well-known West Country consultants on the staff of the Bristol General Hospital. Communicating their observations to the *British Medical Journal* (June 13, 1914), in connection with the thirty-three cases notified up to the end of February of this year from the beginning of 1913, these observers drew attention to the difficulty of early diagnosis. Particularly is this so in infants, where the condition may be mistaken for broncho-pneumonia or tuberculous meningitis. They emphasise the importance of early diagnosis as being essential if the treatment is to be successful. In most of the cases they report as having recovered under intraspinal injections of antimeningococcal serum, treatment was begun within ten days of the commencement of the illness. "No cases recovered which came under treatment late, and the serum seemed to have little or no effect on them." In several cases a leptothrix bacillus was found in association with the meningococcus, but did not appear to have affected the clinical course of the disease.

Dr. Bernard Myers, who has just joined the staff of the Royal Waterloo Hospital for Children and Women, studied medicine at Edinburgh University, St. Bartholomew's Hospital, and at King's College; he is an M.D. Edin., and was elected an M.R.C.P. Lond. two years ago. Lately he has been giving a good deal of attention to the diseases of children, having been working at the Great Ormond Street Hospital, the East London Children's Hospital, and at the "Royal Waterloo." Dr. Myers is one of the physicians at the Western General Dispensary.

Sir Hugh Reeve Beevor, Bart., M.D., F.R.C.P., who has just been appointed consulting physician to King's College Hospital, retired from active work on the medical staff of that institution some little time ago, and has been living in retirement at his place in Norfolk. He was at one time representative for the Society of Apothecaries on the General Medical Council.

